# Association between Alzheimer’s disease and MHC-I antigen processing and presentation pathway: a narrative review

**DOI:** 10.3389/fimmu.2026.1877167

**Published:** 2026-07-10

**Authors:** Caixian Wang, Qilin Yun, Zuoming Zhang, Han Zhao, Fangzhao Lin, Haijun Cao

**Affiliations:** Institute of Blood Transfusion, Chinese Academy of Medical Sciences & Peking Union Medical College, Chengdu, China

**Keywords:** Alzheimer’s disease, antigen presentation, human leukocyte antigen class I, major histocompatibility complex class I, (MHC-I), microglia

## Abstract

Alzheimer’s disease (AD) is the most common cause of dementia worldwide and remains a major public health burden. Although amyloid-beta deposition and tau pathology are the defining pathological features of AD, increasing evidence indicates that immune dysregulation and chronic neuroinflammation also contribute to disease onset and progression. However, the specific immune pathways involved in AD and their mechanistic relevance remain incompletely understood. The major histocompatibility complex class I (MHC-I) antigen processing and presentation pathway has attracted growing attention because of its classical role in adaptive immunity and its potential functions within the central nervous system. In this narrative review, we summarize current evidence linking AD to the MHC-I, or human leukocyte antigen class I (HLA-I), pathway from genetic, molecular, cellular, and immunological perspectives. Available studies implicate the broader HLA region in AD susceptibility and suggest that alterations in HLA-I-related loci, antigen-processing machinery, MHC-I-associated molecules, and downstream immune responses may contribute to disease heterogeneity. At the molecular and cellular levels, changes in MHC-I molecules, antigen-processing machinery, and associated signaling pathways have been reported in microglia, neurons, astrocytes, and oligodendroglial lineage cells. In parallel, changes involving β_2_-microglobulin and the presence of expanded or cytotoxic like CD8^+^ T cells suggest that adaptive immune mechanisms may participate in AD pathology. Nevertheless, direct evidence demonstrating immune responses specific to particular antigens and restricted by HLA-I in human AD remains limited. Overall, current findings indicate that the MHC-I/HLA-I pathway may represent an important component of AD pathophysiology and contribute to disease progression by influencing immune homeostasis and cellular interactions within the central nervous system. Further studies integrating human tissue analysis, immunopeptidomics, spatial profiling, and paired T cell receptor approaches are needed to clarify its mechanistic, biomarker, and therapeutic significance.

## 1 Introduction

Alzheimer’s disease (AD) is a progressive neurodegenerative disorder characterized by irreversible cognitive decline, synaptic dysfunction, and neuronal loss. As the leading cause of dementia worldwide, AD accounts for approximately 60%–70% of all dementia cases (1). According to the World Health Organization, the number of people living with dementia is projected to increase from 55 million in 2019 to 139 million by 2050, while the annual global cost is expected to rise from USD 1.3 trillion in 2019 to USD 2.8 trillion by 2030 (2). This rapidly increasing disease burden underscores the urgent need to better define the biological mechanisms underlying AD and to develop more effective disease-modifying therapies.

For several decades, AD research has focused largely on two defining neuropathological features: the extracellular amyloid-beta (Aβ) plaques and the intracellular aggregation of hyperphosphorylated tau in neurofibrillary tangles (NFTs). These observations gave rise to several influential pathogenic frameworks, among which the amyloid cascade hypothesis has been the most prominent. In its classical form, this hypothesis proposes that an imbalance between Aβ production and clearance initiates a sequence of downstream events, including tau pathology, neuroinflammation, synaptic dysfunction, and neuronal death (3, 4). However, the modest clinical benefits and restricted applicability of amyloid-targeting therapies suggest that AD cannot be fully explained by a single molecular pathway (5–9). Rather, AD is increasingly viewed as a biologically complex disorder influenced by multiple interacting mechanisms.

Among these mechanisms, neuroinflammation has emerged as an important component of AD pathobiology (10–12). Sustained activation of innate immune cells, particularly microglia and astrocytes, together with altered communication between the central and peripheral immune systems, has been linked to synaptic dysfunction and neuronal injury in AD (13, 14). Human genetics studies have further strengthened the relevance of immune mechanisms in AD, particularly for loci related to microglial and immune regulation, including *TREM2* and the human leukocyte antigen (HLA) region (15, 16). In parallel, recent transcriptomic and proteomic studies have revealed that molecular alterations occurring in AD involve immune signaling pathways and the expression of immune functional genes (17, 18). Collectively, these findings support the view that immune dysregulation is embedded in AD biology rather than a mere secondary consequence of neurodegeneration.

Antigen processing and presentation are core components of adaptive immunity (19). The principal function of major histocompatibility complex (MHC) molecules is to present peptide antigens to T lymphocytes. MHC molecules and their encoding genes are classically divided into class I, class II, and class III groups. Classical MHC class I (MHC-I) molecules are expressed on most nucleated cells, although their expression levels vary substantially across cell types. Their canonical function is to present endogenous peptide antigens to CD8^+^ cytotoxic T cells, thereby supporting immune surveillance and elimination of abnormal cells (20).

The central nervous system (CNS) was once regarded as an immune-privileged site, and neurons were generally thought not to express MHC-I under resting conditions (21). This view has been substantially revised. The CNS is increasingly understood as an actively regulated immune environment that communicates bidirectionally with the peripheral immune system, rather than an isolated immune-privileged compartment (13, 22). Accumulating evidence indicates that MHC-I molecules are expressed in the CNS and that their expression levels and cellular localization are dynamically regulated (23, 24). Beyond their canonical role in antigen presentation, MHC-I molecules have been implicated in several aspects of CNS physiology, including synaptic plasticity and pruning, axonal remodeling, and maintenance of neuronal structural integrity and neural homeostasis (25–28). These observations suggest that MHC-I in the CNS cannot be interpreted solely through the framework of peripheral immunology.

Against this background, the MHC-I antigen processing and presentation pathway, and particularly the HLA-I axis, has become an increasingly relevant topic in AD research. Clinical and experimental studies have documented aberrant expression of MHC−I genes and proteins within both the CNS and peripheral immune compartments, alongside evidence regarding β_2_-microglobulin (β_2_M), disrupted neuronal MHC-I function, and immunological changes mediated by CD8^+^ T cells (15, 18–20, 29–36). However, these lines of evidence are not mechanistically equivalent. Genetic association does not by itself establish causality, altered expression does not necessarily indicate productive antigen presentation, and adaptive immune changes do not on their own demonstrate responses specific to particular antigens that are restricted by HLA-I. These distinctions make a critical and integrative evaluation of the current literature necessary.

In this structured narrative review, we synthesize current evidence linking AD to the MHC-I antigen processing and presentation pathway across genetic, molecular, cellular, and immunological levels. The literature was identified through a systematic search, and the study selection process was documented using a Preferred Reporting Items for Systematic Reviews and Meta-Analyses (PRISMA) 2020 flow diagram. The review is organized around genetic associations within the HLA region, molecular perturbations related to class I molecules, CNS changes specific to particular cell types, mechanisms involving β_2_M, and the involvement of CD8^+^ T cells, with emphasis on the strength of evidence, key interpretive boundaries, and major unresolved questions.

## 2 Methods

### 2.1 Review design

**Figure 1:**
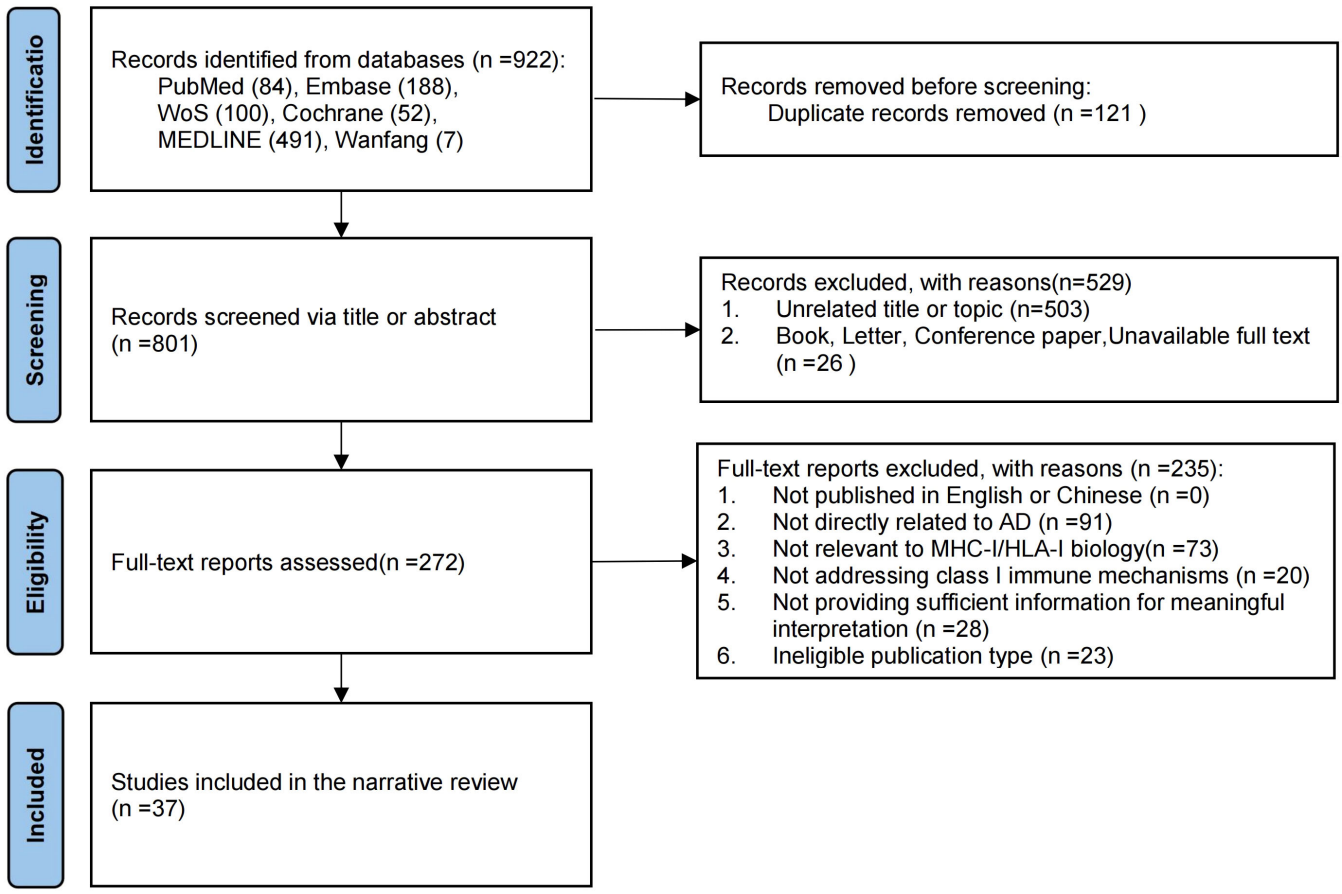
PRISMA 2020 flow diagram of the study selection process.

This study was designed as a structured narrative review informed by a systematic literature search. It aimed to summarize and critically evaluate current evidence linking AD to the MHC-I/HLA-I antigen processing and presentation pathway across genetic, molecular, cellular, and immunological levels. Because the available evidence is heterogeneous in study design, model systems, and outcomes, findings were synthesized narratively and no meta-analysis was performed. To improve transparency, the study identification and selection process was documented using the PRISMA 2020 flow diagram (**Figure 1**) (37).

### 2.2 Information sources and search strategy

Relevant literature was identified through a systematic search of PubMed, Embase, Web of Science, Cochrane Library, MEDLINE, and Wanfang Data from database inception to April 1, 2026. Controlled vocabulary and free-text terms were combined, where appropriate, to identify studies related to AD and the MHC-I/HLA-I antigen processing and presentation pathway. Search terms included, but were not limited to, “Alzheimer’s disease”, “histocompatibility complex class I”, “major histocompatibility complex”, “human leukocyte antigen”, “β_2_-microglobulin”, “antigen processing”, “antigen presentation”, “TAP”, “tapasin”, “immunoproteasome”, “microglia”, “neurons”, “oligodendrocytes”, “CD8^+^ T cells”, and related variants. Search strategies were adapted for each database, and the reference lists of relevant articles were manually screened to identify additional eligible studies. The full search strategies for all databases are provided in the Supplementary Material (**Supplementary Table 1**). Only articles published in English or Chinese were eligible for screening.

### 2.3 Eligibility criteria

Studies were considered eligible if they met at least one of the following criteria: they assessed associations between AD and genetic variation involving MHC-I or HLA-I; examined antigen-processing and presentation machinery associated with MHC-I or HLA-I in AD-relevant tissues, biofluids, or experimental models; evaluated MHC-I or HLA-I changes in specific CNS cell types or barrier-associated compartments; investigated β_2_-microglobulin findings relevant to MHC-I biology in AD; or reported adaptive immune alterations, particularly those involving CD8^+^ T cells, that may inform MHC-I-associated neuroimmune interactions in AD. Human and experimental studies were included when they provided genetic, molecular, cellular, or immunological evidence relevant to AD and MHC-I/HLA-I biology.

Studies were excluded if they were not published in English or Chinese, were not directly related to AD, did not address MHC-I/HLA-I biology or immune mechanisms relevant to class I molecules, lacked sufficient methodological or result details for meaningful interpretation, or were editorials, commentaries, conference abstracts, or duplicate reports. Review articles were not treated as primary evidence, but they were used for conceptual framing and for identifying additional original studies.

### 2.4 Literature selection

All retrieved records were imported into a reference management program, and duplicates were removed before screening. Screening was conducted in two stages: title/abstract screening followed by full-text assessment of potentially eligible articles. Two reviewers (C.W. and Q.Y.) independently screened titles, abstracts and full texts. Disagreements were resolved by discussion or consultation with a third reviewer (H.C.). Reasons for exclusion at the full-text stage were recorded in broad categories. The overall selection process is summarized in the PRISMA 2020 flow diagram.

### 2.5 Data extraction and synthesis

Data were extracted in a structured manner, including study characteristics, participant or model details, experimental conditions where applicable, key outcomes, and main findings related to MHC-I or HLA-I in AD. Additional information on study type, disease context, and major interpretive limitations was also collected. Given the heterogeneity of the included literature, no quantitative pooling was undertaken. Instead, the evidence was synthesized narratively with attention to strength of inference and mechanistic boundaries. The synthesis was organized into major domains, including genetic associations, dysregulation of antigen processing machinery, CNS changes in specific cell types, peripheral immune alterations, mechanisms involving β_2_M, and involvement of CD8^+^ T cells.

## 3 Alzheimer’s disease

### 3.1 Neuropathological characteristics of AD

**Figure 2:**
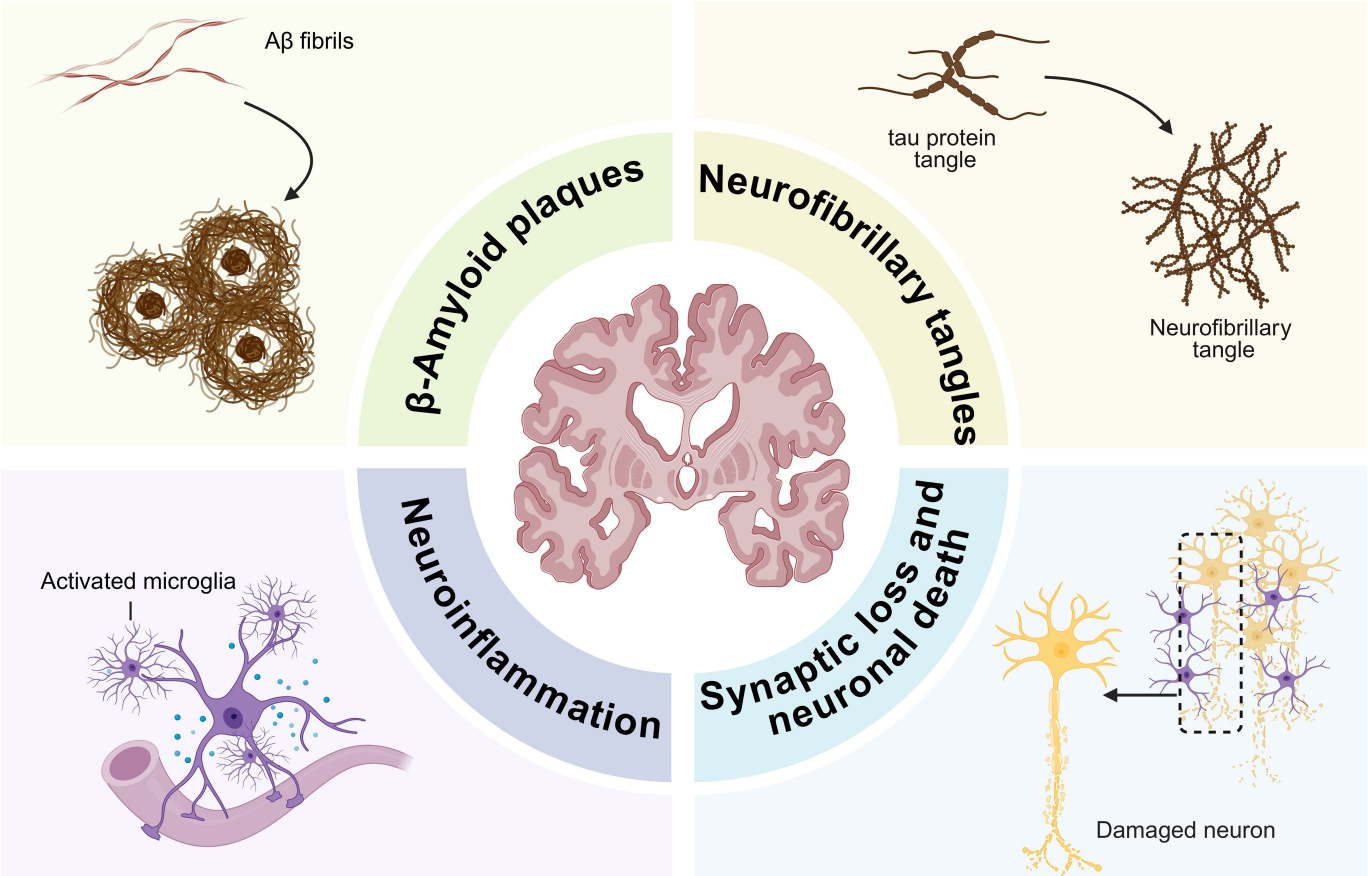
Neuropathological characteristics of AD (Figure created using BioRender.com).

AD is neuropathologically characterized by extracellular Aβ plaques, intracellular NFTs, synaptic degeneration, progressive neuronal loss, and neuroinflammatory changes involving activated microglia and reactive astrocytes (**Figure 2**) (38). Aβ plaques arise from abnormal generation, aggregation, and impaired clearance of Aβ peptides (3, 4, 39, 40), whereas NFTs are composed mainly of hyperphosphorylated tau (p-tau), a microtubule protein that contributes to cytoskeletal stability and axonal transport (41–43). In parallel, microglia and astrocytes respond to protein aggregates, damaged synapses, and disrupted tissue homeostasis through cytokine production, phagocytic activity, and complement-associated inflammatory programs (10–12, 14).

Pathological studies increasingly examine these lesions within a multicellular disease environment rather than as a simple linear cascade of amyloid deposition followed by tau pathology and neuronal loss (44). Aβ oligomers and tau pathology have been linked to synaptic dysfunction, excitatory synapse loss, and network-level propagation of pathology, providing a mechanistic bridge between protein aggregation and neuronal circuit failure (45–52). Single-cell and single-nucleus transcriptomic studies of AD brains have identified disease-associated microglial states, reactive astrocytic programs, and neuronal or synaptic gene changes that vary across brain regions and disease stages (53–55). Experimental AD models further show that microglia, complement activation, and type I interferon signaling can participate in synapse loss and neuroinflammation (56, 57). Together, these findings support a view of AD as a multicellular disorder shaped by interactions among protein aggregation, glial activation, synaptic vulnerability, and neuroimmune signaling.

### 3.2 Immune microenvironment in AD

Recent studies increasingly place the pathological changes of AD within an immune microenvironment (55, 58, 59). In this context, the AD immune microenvironment refers to an integrated tissue-level network composed of CNS-resident cells, vascular and barrier-associated structures, soluble inflammatory mediators, and immune cells in the blood, cerebrospinal fluid, meninges, and brain parenchyma.

The cellular phase model proposed by De Strooper and Karran reframed amyloid and tau pathology as part of a multicellular response involving neurons, microglia, astrocytes, oligodendrocytes, endothelial cells, and peripheral immune compartments, rather than as a cascade driven by a single lesion (31, 55, 58, 60–64). Single-cell and spatial studies have added cellular and anatomical resolution to this framework. Human brain analyses have described disease-associated microglial states, reactive astrocytic programs, neuronal and synaptic transcriptional changes, and vascular cell alterations relevant to barrier function and neurovascular regulation (54, 62, 65–67). Spatial approaches have further mapped molecular and microglial proteomic states within AD tissue architecture (68).

Experimental studies have begun to connect these cellular states with established AD lesions. Aβ exposure can promote lipid droplet-mediated microglial dysfunction through diacylglycerol acyltransferase 2 (69). Human induced pluripotent stem cell (iPSC)-based studies have linked clusterin (CLU) to astrocyte reactivity and microglia-dependent synaptic density (70). NLRP3 inflammasome activation has been associated with tau pathology (71), while complement-dependent microglial mechanisms contribute to early synapse loss in AD models (57). These findings indicate that innate immune and glial responses are closely embedded in AD-associated protein aggregation, synaptic injury, and tissue remodeling.

Adaptive immune alterations have been described in the same tissue context. Human studies reported increased CD8^+^ effector memory T cells in blood, clonally expanded CD8^+^ T cells in cerebrospinal fluid (CSF), and cytotoxic like CD8^+^ T cells in AD-associated brain or meningeal compartments (31, 60, 61, 72, 73). Experimental models have described infiltrating CD8^+^ resident memory T cells at CNS interfaces, stage dependent effects of clonally expanded CD8^+^ T cells on amyloid pathology, and granzyme K^+^ CD8 T cells that interact with microglia in tauopathy settings (74–77). Across these studies, CD8^+^ T-cell phenotypes vary by compartment, pathological context, and disease stage.

### 3.3 Genetic basis of AD

Genetic studies have substantially shaped current understanding of AD biology. Rare familial forms of AD are most associated with pathogenic variants in *APP*, *PSEN1*, or *PSEN2*, which affect amyloid precursor protein processing and Aβ production (78). These high-penetrance variants account for only a small proportion of AD. Most cases occur sporadically and reflect a more complex genetic background involving common susceptibility alleles and rare variants with larger effects.

Among common susceptibility factors, the apolipoprotein E (*APOE*) *ϵ*4 allele remains the most reproducible genetic risk allele for sporadic AD and has been associated with Aβ metabolism, tau-related neurodegeneration, lipid handling, microglial state changes, and inflammatory responses (79–82). Large-scale genome-wide association studies (GWASs), sequencing, and multi-omics studies have extended this genetic framework beyond amyloid processing. Common and rare variants in or near *CLU*, *PICALM*, *BIN1*, *CR1*, *CD33*, *ABCA7*, *TREM2*, *PLCG2*, *ABI3*, *SORL1*, and related loci implicate lipid metabolism, endocytosis, complement signaling, microglial activation, phagocytosis, and innate immune regulation in AD susceptibility (15–17, 44, 55, 83–88).

These findings place immune regulation, glial biology, and neuroinflammatory pathways within the genetic architecture of AD. Although *APOE* remains the strongest and most reproducible common genetic risk factor, signals in immune-related loci, including the broader HLA region, suggest that adaptive and innate immune mechanisms may contribute to AD heterogeneity.

## 4 MHC-I antigen processing and presentation pathway

MHC-I molecules present endogenous peptides to CD8^+^ T cells and thereby support immune surveillance against intracellular infection, malignant transformation, and cellular stress (19, 20, 89). In human, classical MHC-I molecules are encoded by *HLA-A*, *HLA-B*, and *HLA-C.* Each molecule consists of a polymorphic heavy chain associated with β_2_M; the α1 and α2 domains form the peptide-binding groove (PBG), whereas β_2_M supports folding, structural stability, and cell-surface expression (19, 89–91).

**Figure 3:**
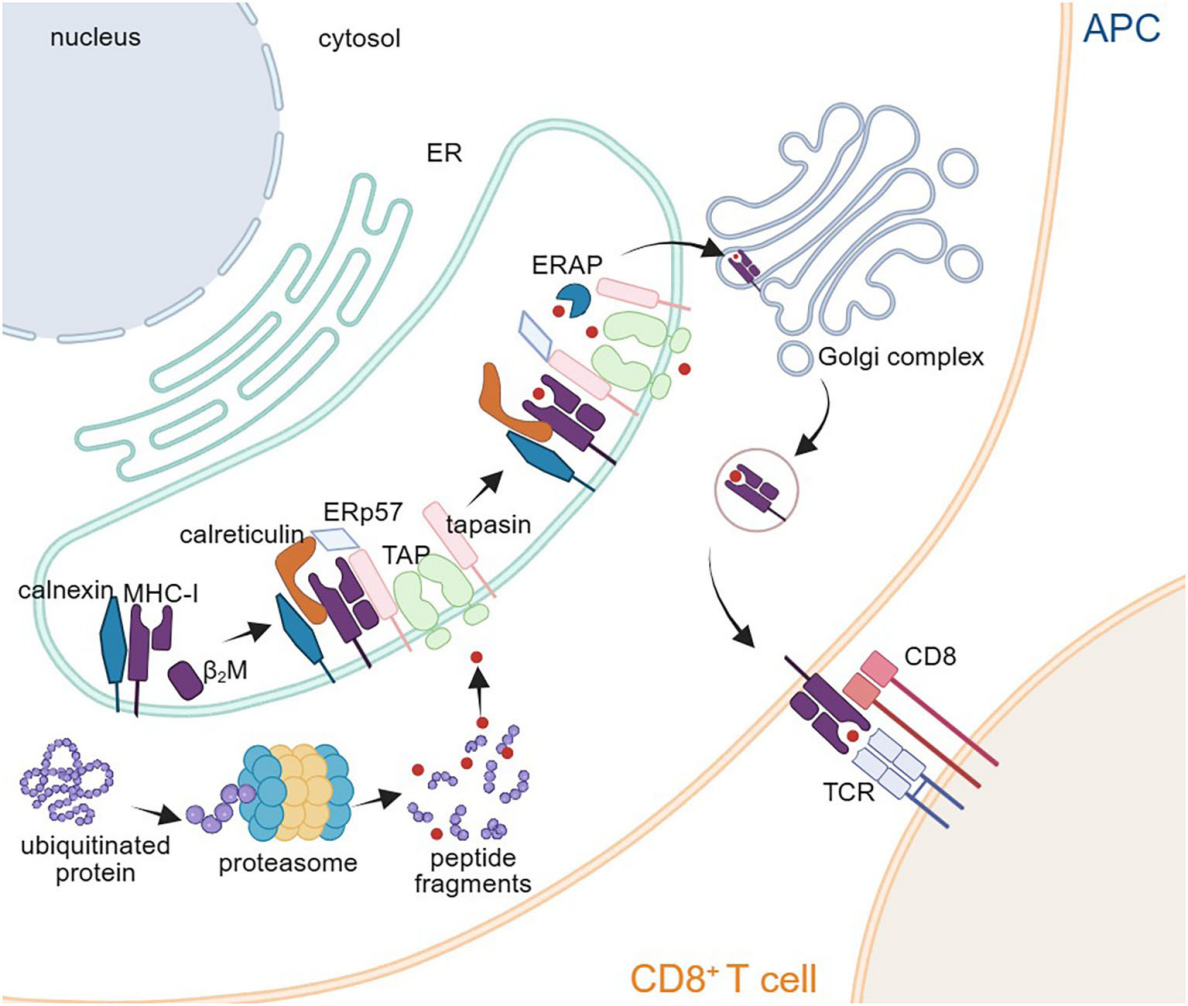
Overview of the MHC-I antigen processing and presentation pathway. Endogenous proteins are degraded by the constitutive proteasome or, under inflammatory conditions, by the immunoproteasome to generate peptide fragments. These peptides are transported into the endoplasmic reticulum (ER) by the TAP, trimmed by endoplasmic reticulum aminopeptidase (ERAP), and loaded onto MHC-I heavy chain and β_2_M complexes with the assistance of the PLC. Stable pMHC-I complexes are then transported through the Golgi apparatus to the cell surface, where they are recognized by T cell receptors (TCRs) on CD8^+^ T cells. (Figure created using BioRender.com)

In the classical MHC-I pathway, cytosolic or nuclear proteins are degraded by the ubiquitin-proteasome system (UPS). Under inflammatory conditions, interferon-γ (IFN-γ) can induce immunoproteasome formation and reshape the peptide repertoire available for class I loading (92, 93). Peptides are then transported into the endoplasmic reticulum by transporter associated with antigen processing (TAP), trimmed by endoplasmic reticulum aminopeptidases, loaded onto MHC-I heavy chain-β_2_M complexes with the assistance of the peptide-loading complex (PLC), and transported through the Golgi apparatus to the cell surface as stable peptide-MHC-I (pMHC-I) complexes (**Figure 3**) (19, 89, 94).

In the nervous system, MHC-I pathway molecules are not restricted to classical immune compartments. Neurons and glial cells generally show low basal MHC-I expression, but class I molecules and related pathway genes can be detected during development and can be induced by neuronal activity, interferon signaling, injury, aging, and disease (24, 36, 95–101). Experimental studies have also linked neuronal MHC-I molecules to synaptic refinement, glutamate receptor regulation, and maintenance of neuronal structural complexity, indicating that class I biology in the CNS extends beyond conventional immune surveillance (25, 27, 28, 102).

Researches on MHC-I biology in the nervous system have moved from documenting CNS expression to defining functional roles. Early work demonstrated activity-regulated class I expression in the developing and adult CNS and showed that class I molecules contribute to activity-dependent synaptic refinement (96, 98). Subsequent studies connected neuronal MHC-I molecules with *N*-Methyl-D-aspartate receptor signaling, α-amino-3-hydroxy-5-methyl-4-isoxazolepropionic acid receptor trafficking, visual cortical synapse organization, and changes in neuronal structural complexity during aging (25, 27, 28, 97). In neurological disease models, MHC-I pathway components have been associated with inflammatory and neurodegenerative processes, including the vulnerability of catecholaminergic neurons to T-cell-mediated injury in a Parkinsonian model and β_2_M-dependent modulation of amyotrophic lateral sclerosis progression (103, 104).

AD studies have added disease-specific evidence, including conserved microglial MHC-I induction across aging and AD models and human tissue, neuronal MHC-I upregulation in *APOE ϵ4* carriers with tau neurodegeneration, and destabilization of neuronal MHC-I-β_2_M complexes by Aβ (33, 34, 36).

## 5 Association between MHC-I antigen processing and presentation pathway and AD

### 5.1 Genetic association of HLA-I loci with AD risk

Genetic studies support the involvement of the broader HLA region in AD susceptibility, but the most consistently replicated signals within this region map predominantly to class II loci rather than to classical HLA-I genes (29, 105). Thus, although HLA-region variation appears to be relevant to AD risk, current genetic evidence does not yet establish classical HLA-I loci as major independent risk determinants (83, 106).

Direct evidence linking classical HLA-I loci to AD has come mainly from candidate-gene studies of *HLA-A* and *HLA-B*, whereas evidence for *HLA-C* has historically been limited (**Table 1**). Reported associations between classical HLA-I alleles and AD risk or age at onset vary substantially by study design, population, and strength of evidence. Studies of *HLA-A* reported associations with disease susceptibility or age at onset in specific cohorts (107, 108). By contrast, findings related to *HLA-B*, especially HLA-B*07, represent historically important signals but have not been sufficiently consistent to establish a robust and reproducible association (109, 110). Earlier reports involving HLA-B*15 and HLA-B*16 should be interpreted as preliminary historical findings rather than strong contemporary evidence (111, 112).

**Table 1:** Representative human studies reporting direct associations between classical HLA-I loci and AD-related risk or age at onset.

HLA-I locus/allele	Study	Population/design	Reported finding
HLA-A*01	Guerini et al., 2009 (107)	Italian case–control study	HLA-A*01 was associated with later age at onset, particularly in late-onset cases
HLA-A alleles (including A02-group and A2402)	Ma et al., 2008 (108)	Southern Chinese community, case–control study	Associations between *HLA-A* alleles and AD were reported, including findings related to susceptibility and age at onset
HLA-B*07	Lehmann et al., 2001 (109)	Confirmed late-onset AD, multi-cohort case-control study	An initial association between HLA-B*07 and late-onset AD was reported, with a stronger signal in *APOE ϵ4*-negative subjects
HLA-B*07	Lehmann et al., 2006 (110)	Replication case-control study	Follow-up analysis further examined the HLA-B*07 signal in AD
HLA-B*15	Renvoize, 1984 (111)	Family/HLA study	Increased frequency of HLA-B*15 was reported in AD patients
HLA-B*16	Endo et al., 1986 (112)	Japanese dementia cohort	An association between HLA-B*16 and senile dementia of Alzheimer type was reported

More recent data suggest that classical HLA-I loci may also contribute to AD-related genetic heterogeneity through interaction with established risk factors. In a UK Biobank-based report, Yashin et al. identified several variants in the *HLA-C* region associated with altered odds of AD in mothers carrying at least one *APOE ϵ4* allele, with both apparently protective and deleterious signals observed (113). This finding suggests that *HLA-C-*related variation may modify the effect of *APOE ϵ4* in a context dependent manner rather than acting as a uniform main-effect risk locus. However, the evidence remains preliminary and requires replication in independent cohorts, particularly given the complex linkage structure of the HLA region and the need for high resolution validation.

Overall, classical HLA-I variation may modify AD heterogeneity, but the available evidence is less robust and less consistently replicated than that reported for HLA-II-related signals within the broader HLA region (83, 85). Classical HLA-I loci should therefore not currently be regarded as established major independent AD risk loci. Instead, they are best viewed as candidate contributors whose effects may depend on ancestry, linkage relationships within the HLA region, or interaction with other disease-relevant factors such as *APOE ϵ4*. Larger multiple-ancestry studies using high-resolution HLA imputation or direct typing will be required to determine whether specific classical *HLA-I* alleles exert reproducible and independent effects on AD susceptibility or age at onset.

### 5.2 Dysregulation of MHC-I antigen processing in AD

#### 5.2.1 UPS and immunoproteasome dysfunction

The proteasome is a major source of endogenous peptides available for MHC-I loading. Under physiological conditions, the UPS degrades unnecessary, misfolded, or damaged intracellular proteins (114). During inflammatory or cytokine-stimulated states, catalytic subunits of the constitutive proteasome can be replaced by inducible subunits to form the immunoproteasome. This remodeling changes substrate specificity and can reshape the peptide repertoire available for class I loading (93, 115). In AD experimental models, including APP/PS1 mice, altered expression of immunoproteasome subunits has been reported (116).

Proteasomal dysfunction and impaired proteostasis have been described in both human AD studies and experimental models (117–120). Disease-associated protein oligomers can inhibit proteasome function in experimental systems, while reactive glial cells in AD contexts have shown increased expression of immunoproteasome subunits and enhanced proteolytic activity (94, 114). These observations are relevant to the MHC-I pathway because they involve the intracellular machinery that generates many class I ligands (119). However, altered proteasome activity or immunoproteasome induction does not establish which peptides are loaded onto HLA-I molecules are displayed in AD tissue.

Proteasome biology in AD is increasingly being examined in more defined cellular, genetic, and disease contexts. Paradise et al. identified the neuron-specific plasma membrane proteasome, or neuroproteasome, as a regulator of endogenous tau proteostasis; selective neuroproteasome inhibition induced *de novo* formation of sarkosyl-insoluble tau paired helical filaments, and neuroproteasome abundance varied with *APOE* isoform and age (121). In a clinical imaging cohort, Koo et al. reported that lower circulating proteasome activity was associated with greater amyloid and tau burden, smaller hippocampal volume, and worse cognition in *APOE* ϵ4 carriers, whereas these associations were not observed in noncarriers (68). In 5xFAD mice, Ouk et al. found that rapamycin reduced CD11c^+^ microglia, decreased immunoproteasome content and activity, and increased amyloid plaque load (122). Together, these studies link proteasome activity to tau assembly, vulnerability in *APOE*, microglial-state changes, and amyloid pathology, but the HLA-I immunopeptidome in AD remains largely undefined.

#### 5.2.2 Impaired autophagy-lysosome system

The autophagy-lysosome system cooperates with the UPS to maintain proteostasis, organelle quality control, and cellular adaptation to stress. Through macroautophagy, microautophagy, and chaperone-mediated autophagy, this system removes cytoplasmic material that is not efficiently handled by proteasomal degradation. In selected cellular contexts, autophagy can also influence antigen availability for MHC-I presentation or cross-presentation (102, 123). In AD, this system is relevant not only as a clearance pathway for misfolded or aggregated proteins but also through its influence on endolysosomal function and inflammatory signaling.

Autophagy-lysosome impairment is well documented in AD. Clinical and experimental studies have reported reduced Beclin 1 expression, blocked autophagic flux, lysosomal dysfunction, and accumulation of undegraded substrates (124, 125). These abnormalities compromise intracellular protein clearance and contribute to the accumulation of Aβ and other abnormal proteins. Whether such changes functionally alter MHC-I peptide loading or presentation in AD remains less clearly defined. Impaired autophagic degradation could plausibly affect intracellular peptide availability, inflammatory signaling, and interactions with proteasomal pathways, but current evidence does not establish a direct causal link between autophagy failure and altered HLA-I antigen presentation in human AD brain tissue (126). Recent studies have bugun to examine this issue within defined AD genetic and experimental settings. Del Ser-Badia et al. analyzed human brain tissue, fibroblasts, and iPSC derived neurons from carriers of PSEN1-associated AD, together with a neuronal model of presenilin deficiency and tauopathy, and reported that presenilin function is required for neuronal tau clearance through autophagy and proteasome pathways (127). More broadly, these findings connect autophagy-lysosome dysfunction with proteasomal activity, microglial phenotype, and amyloid or tau pathology. The direct impact of autophagy-lysosome impairment on HLA-I peptide presentation in AD, however, remains to be demonstrated.

#### 5.2.3 MHC-I complex stability

The mature MHC-I complex consists of a polymorphic heavy chain encoded by *HLA-A*, *HLA-B*, or *HLA-C*, β_2_M, and a bound peptide that stabilizes the PBG and enables cell-surface display (19, 20, 89–91). Few AD studies have examined the intact pMHC-I complex as a biochemical unit. Instead, most evidence comes from measurements of individual components or downstream readouts, including HLA-I heavy-chain transcripts, *B2M* or β_2_M abundance, peptide-loading machinery, surface class I staining, and CD8^+^ T-cell phenotypes. These readouts are informative, but they do not capture the same biological event.

At the level of the MHC-I heavy chain and β_2_M, transcriptomic studies have reported induction of HLA-I genes and *B2M* in AD cellular states, particularly in microglia and inflammatory tissue contexts (33, 53, 54, 56, 103, 128). Kellogg et al. further showed that microglial MHC-I induction during aging and AD is conserved across mouse models and human tissue (33). These findings support altered expression of MHC-I pathway components in AD, although transcript or protein abundance alone does not establish surface pMHC-I formation, peptide cargo, or T cell specificity.

Findings of β_2_M illustrate the need to distinguish MHC-I complex stability from the broader biology of individual pathway components. Kim et al. showed that oligomeric Aβ destabilized the neuronal MHC-I-β_2_M complex, reduced the interaction between MHC-I and neural cell adhesion molecule 1 (NCAM1), and altered downstream neuronal signaling (34). Zhao et al. described a separate process in which β_2_M coaggregated with Aβ and aggravated amyloid pathology and cognitive impairment in AD model mice (129). Both studies implicate β_2_M in AD pathobiology, but only the former directly addresses the stability of an MHC-I complex.

The peptide cargo of MHC-I molecules remains the least characterized component in AD. Immunopeptidomic profiling of human induced pluripotent stem cell-derived microglia has begun to define HLA-bound peptides in a CNS myeloid system, including peptides derived from proteins implicated in neurodegenerative disease biology (130, 131). Such data are closer to the intact pMHC-I complex than transcriptomic induction of HLA-I or *B2M.* Nevertheless, they remain distinct from direct demonstration of antigen-specific CD8^+^ T-cell recognition in AD brain tissue.

### 5.3 MHC-I dysregulation across cell types in the AD brain

#### 5.3.1 Microglia

Within the CNS, microglia are the cell population most consistently associated with MHC-I pathway changes in aging and AD. Ribosome-associated profiling, bulk and sorted transcriptomics, and single-cell or single-nucleus RNA sequencing studies in mouse models and human tissues, microglia have repeated shown increased expression of MHC-I pathway genes, including *B2M*, class I heavy-chain genes, and antigen-processing components such as Tap1, Tap2, and tapasin (TAPBP) (33, 53, 54, 56, 103, 128). In AD, these changes are often enriched in disease-associated microglial states or plaque-associated subsets, suggesting that class I pathway induction is linked to immune activation rather than being a uniform feature of all microglia (66).

This pattern is important for two reasons. First, microglial MHC-I upregulation appears to be a reproducible component of the AD immune landscape. Second, activation of the MHC-I pathway in microglia may provide a molecular basis for local interactions with adaptive immune cells, particularly CD8^+^ T cells, in neuroinflammatory settings associated with AD and tauopathy (31, 75, 132, 133). Experimental studies support the capacity of microglia to acquire peptide-processing and antigen-display functions under inflammatory conditions. For example, immunopeptidomic analysis of IFN-γ-stimulated induced pluripotent stem cell-derived microglia-like cells revealed a broad repertoire of HLA-I-bound peptides. Notably, this repertoire included peptides derived from tau and from several AD-associated proteins (134). These datas indicate that, under defined experimental conditions, microglia can generate and display class I-associated peptide repertoires relevant to neurodegenerative disease biology (131).

The interpretive boundary here is crucial. Although experimental evidence indicates that microglia can acquire MHC-I antigen-presenting or cross-presenting capacity under inflammatory or infectious conditions (134), these findings should be distinguished from direct evidence that microglia present defined AD-relevant peptides to CD8^+^ T cells in human brain tissue (31, 133). Likewise, increased expression of the MHC-I machinery in microglia does not, by itself, establish that a complete and functionally effective antigen-presenting process is occurring *in vivo*. At present, the strongest conclusion supported by the literature is that microglia in AD show robust activation of molecular programs related to MHC-I, providing a plausible basis for interactions with adaptive immune cells (29, 30, 33). Whether such interactions are specific to particular antigens, persist across disease stages, and exert primarily pathogenic, compensatory, or mixed effects remains unresolved.

This distinction is particularly important when considering the relationship between microglia and infiltrating CD8^+^ T cells. Several studies in AD models and related neuroinflammatory settings suggest that induction of MHC-I in cells of the myeloid lineage may facilitate T-cell recruitment or retention (32, 74, 134). Nevertheless, much of this evidence comes from model systems, inflammatory perturbations, or disease contexts other than AD. Accordingly, microglial MHC-I upregulation should be regarded as a marker of immune activation and possible neuroimmune crosstalk, rather than direct evidence of a defined antigen-presentation axis linking microglia to CD8^+^ T cells in human AD.

#### 5.3.2 Neurons

Neuronal MHC-I dysregulation is conceptually important in AD because it lies at the intersection of neurodegeneration, synaptic biology, and immune signaling. Under physiological conditions, neuronal MHC-I expression is generally low, although neurons can express MHC-I under conditions of stress, inflammation, or injury (26, 98, 99, 101). In AD, the relevance of neuronal MHC-I is not limited to potential antigen presentation. It also concerns the possibility that changes related to class I molecules in neurons may alter synaptic homeostasis, structural integrity, and vulnerability to injury.

A major line of evidence comes from work showing that neuronal *APOE ϵ4* can induce MHC-I upregulation and is associated with enhanced tau-related neurodegeneration (36). This finding connects a major AD genetic risk factor to neuronal regulation of a classical class I pathway component. It also suggests that neuronal MHC-I upregulation in AD may arise not only as a secondary consequence of inflammation but also through genotype-dependent intrinsic signaling programs. In parallel, additional studies have shown that Aβ oligomers can destabilize the neuronal MHC-I-β_2_M complex and interfere with its interaction with neuronal signaling partners such as NCAM1, thereby altering neuronal functions associated with MHC-I (34). These observations point to two separable aspects of neuronal MHC-I dysregulation in AD: increased expression and compromised complex stability or signaling function.

This distinction complicates any attempt to interpret neuronal MHC-I exclusively in terms of classical immune function. In neurons, elevated MHC-I expression does not necessarily mean that the cell has adopted a conventional antigen-presenting phenotype. It may instead reflect altered synaptic signaling, stress adaptation, aberrant interactions with glia, or increased susceptibility to immune-mediated damage. Similarly, destabilization of the neuronal MHC-I complex by Aβ points to disruption of homeostatic neuronal functions that may be only partly related to antigen presentation (34). Thus, neuronal MHC-I should not be reduced to a simple immune marker in AD.

At the same time, the possibility that neuronal MHC-I influences interactions with CD8^+^ T cells cannot be dismissed. In other neurodegenerative or inflammatory settings, neuronal MHC-I has been linked to increased immune vulnerability (26, 135). In AD, the coexistence of neuronal stress, elevated neuronal MHC-I, and infiltrating CD8^+^ T cells provides a biologically plausible framework for immune-mediated injury. Yet direct evidence that neurons in human AD act as bona fide antigen-presenting targets for recognition by antigen-specific CD8^+^ T cells remains limited (31, 32, 36, 73). The current literature therefore supports a more cautious conclusion: dysregulation of neuronal MHC-I is likely to be biologically relevant in AD, but its primary significance may lie in altered neuronal homeostasis and vulnerability rather than in established classical antigen presentation.

#### 5.3.3 Astrocytes and oligodendrocytes

Astrocytes are prominent participants in the cellular response to AD, with roles in synaptic regulation, metabolic support, blood-brain barrier interactions, cytokine and chemokine signaling, and communication with microglia and immune cells. Single-cell and single-nucleus studies have identified distinct astrocyte states in AD, and iPSC-derived astrocyte models have linked genetic risk and resilience to changes in extracellular matrix organization, intracellular trafficking, mitochondrial function, fatty acid oxidation, and interferon response programs (54, 136, 137). Mechanistic studies further implicate astrocyte reactivity in CLU-dependent regulation of microglia-mediated synaptic changes and in GADD45G-driven reactive gliosis and neurodegeneration in AD models (70, 138).

Direct evidence for astrocytic MHC-I expression comes mainly from inflammatory CNS settings rather than from AD tissue itself. In viral encephalomyelitis, astrocytes upregulated surface MHC-I *in vivo*, with kinetics distinct from those of microglia (139, 140). Astrocytic MHC-I expression after systemic immune activation altered microglial activation, neuronal structure, and behavior in mice (141). These studies indicate that astrocytes can activate MHC-I pathway programs under inflammatory pressure, but they do not establish HLA-I-restricted antigen presentation by astrocytes in AD.

By contrast, the more direct astrocyte evidence for antigen-presentation biology currently lies closer to MHC-II and CD4^+^ T cell contexts. In Parkinson’s disease tissue and human astrocyte cultures, astrocytes displayed MHC-II antigen-presentation features and were associated with T cells (142). In AD, Beretta et al. reported that astrocytic lipid droplets contained MHC-II and were linked to trafficking pathways associated with antigen presentation; astrocytes were also found near infiltrating CD4^+^ T cells in AD brain tissue (143). This literature supports the inclusion of astrocytes in analyses of CNS antigen-presentation programs, while also indicating that the strongest astrocyte evidence at present is not equivalent to direct HLA-I presentation to CD8^+^ T cells.

Evidence involving oligodendrocytes is more limited and largely indirect. White matter abnormalities, myelin disruption, and oligodendrocyte stress are increasingly recognized as components of AD pathophysiology, and distinct oligodendrocyte states have been proposed to contribute to tissue remodeling during neurodegeneration (144, 145). Studies in aging and inflammatory disease contexts have shown that CD8^+^ T cells can induce interferon responsive programs in oligodendrocytes and microglia in white matter, and that oligodendrocyte precursor cells can present antigen and serve as cytotoxic targets in inflammatory demyelination (146, 147). These observations support consideration of antigen-processing pathway in oligodendrocyte-lineage cells, but they do not establish functional HLA-I antigen presentation by oligodendrocytes in AD.

### 5.4 MHC-I alterations in the peripheral immune system in AD

Peripheral evidence linking MHC-I biology to AD is derived mainly from whole-blood and peripheral blood mononuclear cell (PBMC) studies. Single-cell RNA sequencing of peripheral blood has identified immune signatures involving monocyte programs and T cell subsets in AD (148), and Duan et al. reported an increase in peripheral blood GZMK^+^ CD8^+^ T cells as a candidate biomarker (149). Altered T cell reactivity has also been reported in the early stages of AD (72). These findings are relevant because monocytes and other nucleated blood cells express HLA-I, whereas CD8^+^ T cells are the principal T cell population that surveys peptide-HLA-I complexes. Nonetheless, most peripheral studies measure immune-cell composition, transcriptional states, interferon responsiveness, or cytotoxic markers rather than loaded HLA-I peptides or antigen-specific CD8^+^ T-cell recognition.

Broader peripheral immune profiling has also connected AD with systemic immune remodeling. Sirkis et al. described highly interferon responsive T cells in PBMCs from patients with early AD, while blood multi-omics analyses have linked peripheral immune genes, epigenetic signals, immune-cell proportions, CSF biomarkers, cognition, and disease progression (150, 151). Findings involving *APOE* genotype, CD4^+^ T cells, or helper T-cell phenotypes may inform the wider immunology of AD, but they should not be read as direct evidence for peripheral MHC-I antigen presentation (152–154).

### 5.5 CD8^+^ T-cell infiltration and retention in the AD brain

CD8^+^ T cells are the adaptive immune population most directly linked to MHC-I biology. In AD, evidence for their involvement spans multiple anatomical compartments rather than a single tissue source. CSF studies have identified clonally expanded CD8^+^ T cells (31), whereas postmortem and single-cell analyses have described CD8^+^ T cells in perivascular regions, meninges, hippocampus, and cortex, often with effector-memory, tissue-retention, terminally differentiated effector memory T cells re-expressing CD45RA (TEMRA), or granzyme signatures (32, 60, 73, 74, 155–158). This compartmental distribution is important because CD8^+^ T-cell accumulation in CSF, CNS borders, or brain parenchyma does not necessarily reflect the same local MHC-I process. Functional studies in AD models have produced a more complex picture. Functional studies in AD models have produced a more complex picture. Mason et al. reported that GZMK^+^ CD8^+^ T cells targeted microglia in tauopathy models and human p-tau lesions, and that CD8^+^ T-cell deletion accelerated p-tau spread and neurological decline in mice (76). Terrabuio et al. reported that ablation of brain CD103^–^CD8^+^ T cells in 3xTg-AD mice ameliorated cognitive decline and reduced neuropathology; they also showed that granzyme K induced neuronal dysfunction and tau hyperphosphorylation in human and mouse cells via protease-activated receptor-1, which is expressed at higher levels in the AD brain (156). In an amyloidosis model, Wang et al. reported that CD8^+^ T cells exacerbated AD-like symptoms (155). Ohyagi et al. found that depletion of clonally expanded CD8^+^ T cells had different effects in AD mice according to disease stage, reducing amyloid plaque accumulation early but worsening amyloid pathology later (75). Gellrich et al. used an Nr4a1 reporter strategy to detect antigen-dependent activation among CD8^+^ and double-negative T cells in APP/PS1 mice, and Kang et al. described CD8^+^ T cells with resident-memory features together with altered choroid plexus epithelial programs in 5xFAD mice (77, 159).

Together, these studies place CD8^+^ T cells within AD immune responses, but they do not support a uniform effector model. The consequences of CD8^+^ T cell activity appear to vary according to disease model, disease stage, anatomical compartment, and T-cell phenotype. For the MHC-I pathway, the central distinction remains whether a study shows CD8^+^ T cell accumulation or activation, or whether it demonstrates HLA-I-restricted recognition of defined AD-relevant peptides. Clonal expansion, cytotoxic programs, tissue retention, and reporter-based activation strengthen the evidence for adaptive immune involvement. Even so, antigen identity, HLA-I restriction, priming site, and target cell type remain unresolved in most AD studies (31, 76, 160).

## 6 Discussion

The evidence reviewed here suggests that MHC-I biology is involved in AD as part of a broader process of neuroimmune remodeling. Such involvement is unlikely to follow a simple linear sequence in which amyloid or tau pathology directly induces classical antigen presentation and then drives a uniform CD8^+^ T-cell response. Rather, MHC-I-related changes appear to arise within an AD tissue environment shaped by proteostatic stress, neuroinflammation, altered CNS cell states, HLA genetic variation, and adaptive immune participation. Such a framework helps reconcile the diverse evidence currently available: the pathway is repeatedly implicated across genetic, molecular, cellular, and immunological levels, but a complete antigen-specific HLA-I-restricted mechanism remains incompletely defined.

One reason MHC-I biology is relevant to AD is that this pathway is closely connected to intracellular protein handling and inflammatory signaling, both of which are profoundly disturbed in AD. Aβ and tau abnormalities are associated with impaired proteostasis, and the proteasome is a major source of endogenous peptides for MHC-I loading (19, 20, 89, 94). Altered proteasome or immunoproteasome activity could therefore influence the peptide pool available for HLA-I presentation. In parallel, neuroinflammation provides a cytokine-rich environment that can induce MHC-I pathway genes, promote immunoproteasome formation, and reshape interactions between CNS-resident cells and infiltrating or border-associated immune cells. Autophagy-lysosome dysfunction may further modify this environment by affecting aggregate clearance, endolysosomal trafficking, organelle turnover, and inflammatory signaling (122, 124–127). β_2_M provides another link between AD pathology and MHC-I biology. Within the MHC-I complex, β_2_M supports folding, stability, and surface expression; outside this structural context, it may influence amyloid pathology through other mechanisms (34, 90, 91, 129). In this setting, AD-associated disturbances in proteostasis and neuroinflammation create a molecular environment in which MHC-I antigen processing, peptide loading, and complex stability could plausibly be altered.

**Figure 4:**
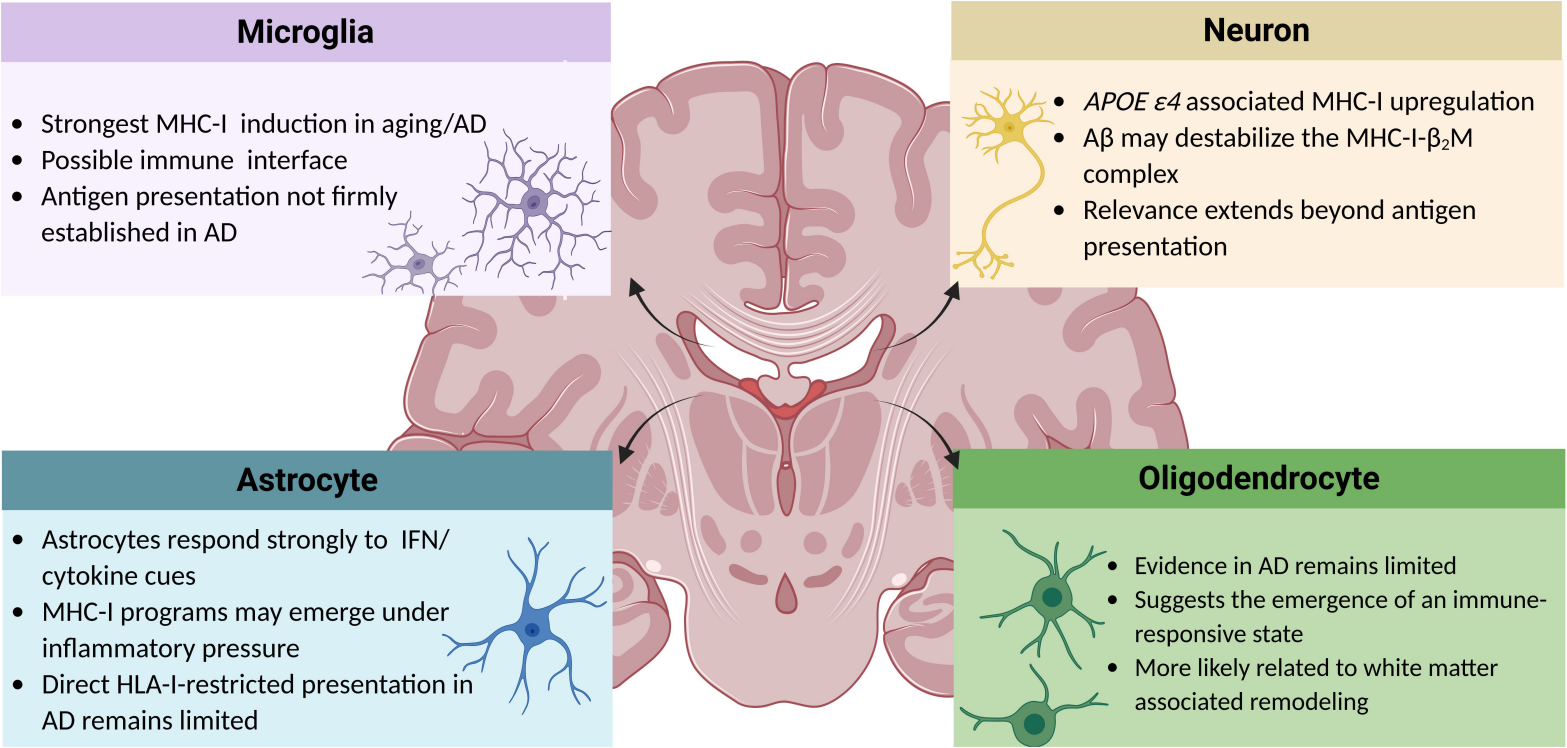
Cellular evidence of changes related to MHC-I in AD. (Figure created using BioRender.com).

Whether these molecular and inflammatory changes become biologically meaningful depends strongly on cellular context. Microglia provide the most consistent evidence for MHC-I pathway induction in aging and AD, particularly in disease-associated or plaque-associated states (33, 62, 67, 136). Their proximity to protein aggregates, inflammatory mediators, and infiltrating or border-associated immune cells makes them plausible local mediators of MHC-I-related neuroimmune interactions. Neurons add a different dimension. In neuronal cells, MHC-I molecules may participate not only in immune recognition but also in synaptic regulation, structural maintenance, stress signaling, and complex stability (25, 27, 28, 34, 36, 98). Astrocytes and oligodendrocytes broaden this cellular landscape, although direct evidence for HLA-I-restricted antigen presentation by these cells in AD remains limited (137, 142, 143, 145) (**Figure 4**). Current data therefore suggest that AD does not contain a uniform MHC-I mechanism across the CNS. Instead, MHC-I-related changes appear to reflect cell-specific responses within a shared inflammatory and proteostatic environment.

CD8^+^ T-cell involvement brings this discussion closer to adaptive immune function. CD8^+^ T cells are the immune population most directly linked to MHC-I biology, and studies have described clonally expanded CD8^+^ T cells in CSF as well as CD8^+^ T cells with effector-memory, cytotoxic, tissue-retention, or granzyme-associated signatures in meningeal, perivascular, hippocampal, and cortical compartments (31, 32, 60, 61, 72, 73, 156–158). Experimental models further indicate that CD8^+^ T-cell effects may vary according to disease stage, anatomical location, and the predominance of amyloid or tau pathology. These findings make an MHC-I-related adaptive immune interface biologically plausible. Yet the presence, expansion, or cytotoxic phenotype of CD8^+^ T cells does not by itself identify the relevant antigen, HLA-I restriction element, priming site, or target cell. The available evidence therefore supports adaptive immune involvement, but not yet a complete antigen-specific MHC-I mechanism.

Spatial context is particularly important because CD8^+^ T-cell localization in AD spans several compartments. Peripheral blood, CSF, meninges, perivascular spaces, and brain parenchyma represent different anatomical and immunological contexts. Entry of CD8^+^ T cells into CNS compartments generally requires adhesion, chemokine sensing, endothelial crawling, and passage across blood-brain or blood-CSF barriers rather than passive diffusion (161). MHC-I upregulation should therefore not be interpreted as a signal that recruits CD8^+^ T cells into the brain. A more likely scenario is that inflammatory cues in AD induce both trafficking-related programs and MHC-I pathway molecules within the same tissue environment. Once CD8^+^ T cells are present in CNS-border or parenchymal compartments, local pMHC-I interactions on endothelial, perivascular, myeloid, glial, or neuronal cells could contribute to their retention, activation, or target recognition. Defining where this occurs will be essential for distinguishing systemic immune activation, CNS-border surveillance, and true parenchymal antigen recognition.

HLA genetic background may further shape whether MHC-I-related immune responses are harmful, neutral, or protective. The HLA region is among the most polymorphic and linkage-dense regions of the human genome, making causal interpretation particularly challenging. *HLA-A*, *HLA-B*, and *HLA-C* differ markedly across populations in allele frequency, haplotype structure, linkage disequilibrium, and peptide-binding repertoire (19, 20, 89). Such features may partly explain why reported classical HLA-I associations with AD have been difficult to reproduce across cohorts. An apparent HLA-I signal may reflect the effect of a specific HLA-I allele, linkage with nearby HLA-II or class III variants, ancestry-specific haplotypes, interaction with *APOE ϵ4*, or differences in HLA typing and imputation resolution (15, 106, 113). Accordingly, inconsistent HLA-I findings should not necessarily be interpreted as evidence against biological relevance, but rather as a reflection of the genetic and immunological complexity of this region.

The genetic architecture of the HLA region may also explain why AD genetics has so far focused more strongly on HLA-II than on HLA-I. The most reproducible HLA-region signals in AD have often mapped to class II loci or class II-related haplotypes, which may be more readily detected in genome-wide association or imputation-based studies and may align more directly with known antigen-presenting-cell and microglial biology (106, 162–164). Notably, some HLA-II alleles or haplotypes have been reported to show protective associations in AD, including findings involving *HLA-DQ* or HLA-DR/DRB*1-related variation (162–164). This observation is conceptually important because it suggests that adaptive immune genetics may influence resilience as well as risk. By analogy, specific HLA-I alleles may also modify AD biology in deleterious or protective directions by shaping peptide-binding repertoires, immune recognition thresholds, and CD8^+^ T-cell interactions. Nevertheless, this possibility has not been adequately tested, partly because HLA-I effects may depend on cellular source, peptide cargo, disease stage, ancestry background, and local immune context.

The central gap in the field is not whether MHC-I pathway alterations occur in AD, but whether these alterations form a complete antigen-specific HLA-I-restricted axis. Most current studies capture only one part of this pathway. Genetic studies do not identify peptide cargo; transcriptomic studies do not demonstrate surface pMHC-I display; immunostaining does not define HLA-bound peptides; T-cell clonality does not establish antigen specificity; and peripheral blood findings do not localize immune events to the CNS (31, 33, 61, 131, 148). Accordingly, alterations in MHC-I pathway genes or proteins should be interpreted as evidence of a relevant neuroimmune program rather than as direct proof of productive antigen presentation.

Addressing this gap will require connecting multiple levels of evidence within the same biological setting. For HLA-I genetics specifically, larger multi-ancestry cohorts, direct high-resolution HLA typing or validated HLA imputation, careful modeling of local linkage disequilibrium, and stratification by APOE genotype, ancestry, and disease stage will be required. Genetic association should also be integrated with functional data, including HLA-I expression, immunopeptidomic profiling, and CD8^+^ T-cell receptor analyses. More broadly, spatial and single-cell transcriptomics can identify the cellular sources and tissue neighborhoods of MHC-I pathway induction; proteomics and immunopeptidomics can define loaded HLA-I peptides; T-cell receptor sequencing can characterize clonal architecture; and paired functional assays can test antigen recognition and target-cell specificity (62, 67, 130, 131, 136). Longitudinal integration with blood, CSF, imaging, and neuropathology will also be important, because MHC-I-related changes may have different meanings across preclinical AD, mild cognitive impairment, and dementia.

The same evidential limits constrain translational interpretation. Current evidence does not support broad inhibition or activation of the MHC-I pathway in AD. Class I molecules support immune surveillance and may also contribute to CNS homeostatic functions, whereas CD8^+^ T-cell effects appear to vary by disease stage, compartment, and target cell (19, 20, 27, 75–77, 98, 155, 159). The more immediate value of this field may lie in disease stratification: identifying AD subgroups marked by proteostatic stress, neuroinflammation, interferon activity, microglial MHC-I pathway induction, β_2_M complex changes, peripheral immune remodeling, CD8^+^ T-cell signatures, or specific HLA backgrounds. These features would become more informative if they could be linked to peptide repertoires, HLA-I restriction, and T-cell specificity.

Overall, MHC-I/HLA-I biology should be viewed as a biologically relevant component of AD-associated neuroimmune remodeling rather than as an established antigen-specific cytotoxic mechanism. The strongest evidence currently supports convergence among proteostatic stress, neuroinflammation, cell-context-dependent MHC-I pathway changes, HLA genetic background, and CD8^+^ T-cell involvement. The key unresolved question is whether these converging signals form a defined antigen-presentation axis in human AD, with identifiable peptide cargo, HLA-I restriction, CD8^+^ T-cell specificity, trafficking route, and cellular target. Resolving this question will determine whether MHC-I biology remains an associated neuroimmune signature or becomes a mechanistically defined component of AD heterogeneity.

## 7 Conclusion

Current evidence indicates that the MHC-I/HLA-I antigen processing and presentation pathway is relevant to AD, but its significance differs across genetic, molecular, cellular, and immunological levels. The strongest support currently lies in the involvement of the HLA region, perturbation of molecular machinery related to class I molecules, and dysregulation across specific CNS cell types, particularly microglia and neurons. By contrast, direct evidence for productive immune responses specific to particular antigens and restricted by HLA-I in human AD remains limited (19, 29).

The MHC-I/HLA-I axis is therefore best viewed as a biologically relevant component of neuroimmune remodeling associated with AD rather than as a fully established pathogenic mechanism. Emerging preclinical findings also suggest possible therapeutic relevance, but current evidence remains insufficient to support broad intervention directed at this pathway. Future studies integrating spatial and single-cell transcriptomics, proteomics, immunopeptidomics, and paired TCR analyses based on human tissue will be essential for clarifying the mechanistic, biomarker, and translational significance of alterations related to MHC-I/HLA-I in AD (62, 67, 130, 131, 136).
