# Supplementary material for: Association between Alzheimer’s disease and MHC-I antigen processing and presentation pathway: a narrative review
Source: Front Immunol. 2026 Jul 10;17:1877167. doi: 10.3389/fimmu.2026.1877167 (PMC13395697; doi:10.3389/fimmu.2026.1877167)
Supplement: Supplementary file 1 [file Table1.docx]

Supplementary Material

# Supplementary Figures and Tables

## Supplementary Tables

| **Supplementary Table S1. Search strategies used for each database** | |
| --- | --- |
| **Database** | **Search strategy** |
| PubMed | (("Alzheimer disease" OR "Alzheimer's disease" OR Alzheimer* OR "Alzheimer dementia")) AND (("major histocompatibility complex class I" OR "MHC class I" OR "MHC-Ⅰ" OR "MHC I" OR "human leukocyte antigen class I" OR "HLA class I" OR "HLA-Ⅰ" OR "HLA I" OR "HLA-A" OR "HLA-B" OR "HLA-C" OR B2M OR "beta-2 microglobulin" OR "β_2_-microglobulin" OR "antigen processing" OR "antigen presentation" OR "peptide-MHC" OR pMHC OR TAP1 OR TAP2 OR "transporter associated with antigen processing" OR tapasin OR TAPBP OR ERAP1 OR ERAP2 OR "endoplasmic reticulum aminopeptidase" OR "peptide-loading complex" OR immunoproteasome OR proteasome OR LMP2 OR LMP7 OR "MECL-1" OR "CD8 T cell" OR "CD8+ T cell" OR "CD8-positive T cell" OR "cytotoxic T cell")) Field: All Fields. Terms may also be entered explicitly as [All Fields] if required by the search interface. |
| Embase | ("Alzheimer disease" OR "Alzheimer's disease" OR Alzheimer* OR "Alzheimer dementia") AND ("major histocompatibility complex class I" OR "MHC class I" OR "MHC-Ⅰ" OR "MHC I" OR "human leukocyte antigen class I" OR "HLA class I" OR "HLA-Ⅰ" OR "HLA I" OR "HLA-A" OR "HLA-B" OR "HLA-C" OR B2M OR "beta-2 microglobulin" OR "β_2_-microglobulin" OR "antigen processing" OR "antigen presentation" OR "peptide-MHC" OR pMHC OR TAP1 OR TAP2 OR "transporter associated with antigen processing" OR tapasin OR TAPBP OR ERAP1 OR ERAP2 OR "endoplasmic reticulum aminopeptidase" OR "peptide-loading complex" OR immunoproteasome OR proteasome OR LMP2 OR LMP7 OR "MECL-1" OR "CD8 T cell" OR "CD8+ T cell" OR "CD8-positive T cell" OR "cytotoxic T cell") Field: All fields/no field restriction in Embase.com. If searched through Ovid Embase, use .af. for each term block. |
| Web of Science | ALL=("Alzheimer disease" OR "Alzheimer's disease" OR Alzheimer* OR "Alzheimer dementia") AND ALL=("major histocompatibility complex class I" OR "MHC class I" OR "MHC-Ⅰ" OR "MHC I" OR "human leukocyte antigen class I" OR "HLA class I" OR "HLA-Ⅰ" OR "HLA I" OR "HLA-A" OR "HLA-B" OR "HLA-C" OR B2M OR "beta-2 microglobulin" OR "β_2_-microglobulin" OR "antigen processing" OR "antigen presentation" OR "peptide-MHC" OR pMHC OR TAP1 OR TAP2 OR "transporter associated with antigen processing" OR tapasin OR TAPBP OR ERAP1 OR ERAP2 OR "endoplasmic reticulum aminopeptidase" OR "peptide-loading complex" OR immunoproteasome OR proteasome OR LMP2 OR LMP7 OR "MECL-1" OR "CD8 T cell" OR "CD8+ T cell" OR "CD8-positive T cell" OR "cytotoxic T cell") Field: All Fields. |
| Cochrane Library | ("Alzheimer disease" OR "Alzheimer's disease" OR Alzheimer* OR "Alzheimer dementia") AND ("major histocompatibility complex class I" OR "MHC class I" OR "MHC-Ⅰ" OR "MHC I" OR "human leukocyte antigen class I" OR "HLA class I" OR "HLA-Ⅰ" OR "HLA I" OR "HLA-A" OR "HLA-B" OR "HLA-C" OR B2M OR "beta-2 microglobulin" OR "β_2_-microglobulin" OR "antigen processing" OR "antigen presentation" OR "peptide-MHC" OR pMHC OR TAP1 OR TAP2 OR "transporter associated with antigen processing" OR tapasin OR TAPBP OR ERAP1 OR ERAP2 OR "endoplasmic reticulum aminopeptidase" OR "peptide-loading complex" OR immunoproteasome OR proteasome OR LMP2 OR LMP7 OR "MECL-1" OR "CD8 T cell" OR "CD8+ T cell" OR "CD8-positive T cell" OR "cytotoxic T cell") Field: All text/all fields. No field label was applied. |
| MEDLINE | 1. ("Alzheimer disease" OR "Alzheimer's disease" OR Alzheimer* OR "Alzheimer dementia").af. 2. ("major histocompatibility complex class I" OR "MHC class I" OR "MHC-Ⅰ" OR "MHC I" OR "human leukocyte antigen class I" OR "HLA class I" OR "HLA-Ⅰ" OR "HLA I" OR "HLA-A" OR "HLA-B" OR "HLA-C" OR B2M OR "beta-2 microglobulin" OR "β_2_-microglobulin" OR "antigen processing" OR "antigen presentation" OR "peptide-MHC" OR pMHC OR TAP1 OR TAP2 OR "transporter associated with antigen processing" OR tapasin OR TAPBP OR ERAP1 OR ERAP2 OR "endoplasmic reticulum aminopeptidase" OR "peptide-loading complex" OR immunoproteasome OR proteasome OR LMP2 OR LMP7 OR "MECL-1" OR "CD8 T cell" OR "CD8+ T cell" OR "CD8-positive T cell" OR "cytotoxic T cell").af. 3. 1 AND 2 4. limit 3 to (English or Chinese) Field: All Fields (.af.). |
| 万方数据库 | ("阿尔茨海默病" OR "阿尔茨海默症" OR "老年痴呆" OR "阿尔茨海默型痴呆") AND ("主要组织相容性复合体Ⅰ类" OR "主要组织相容性复合体I类" OR "MHC-Ⅰ" OR "MHC I" OR "人类白细胞抗原Ⅰ类" OR "人类白细胞抗原I类" OR "HLA-Ⅰ" OR "HLA I" OR "HLA-A" OR "HLA-B" OR "HLA-C" OR "β2微球蛋白" OR "β2-微球蛋白" OR "B2M" OR "抗原加工" OR "抗原处理" OR "抗原呈递" OR "抗原提呈" OR "TAP1" OR "TAP2" OR "抗原加工相关转运体" OR "tapasin" OR "TAPBP" OR "ERAP1" OR "ERAP2" OR "内质网氨肽酶" OR "肽装载复合体" OR "免疫蛋白酶体" OR "蛋白酶体" OR "CD8 T细胞" OR "CD8阳性T细胞" OR "细胞毒性T细胞") 字段：全部字段/All Fields。 |

**Search date range:** from database inception to April 1, 2026. All searches used free combinations of Alzheimer’s disease terms and MHC-Ⅰ/HLA-Ⅰ antigen processing and presentation pathway terms.

Note. The original database search covered records available from database inception to April 1, 2026. Additional targeted searches and citation tracking were performed during revision to address reviewer comments and update topics expanded in the revised manuscript. Records first available after April 1, 2026 were used only to refine the narrative synthesis and were not incorporated into the original PRISMA flow diagram.
